# *N*′-(5-Chloro-2-hydroxy­benzyl­idene)-3,4,5-trihydroxy­benzohydrazide dihydrate

**DOI:** 10.1107/S1600536809010812

**Published:** 2009-03-28

**Authors:** Abeer A. Abdul Alhadi, Hapipah Mohd. Ali, Seik Weng Ng

**Affiliations:** a Department of Chemistry, University of Malaya, 50603 Kuala Lumpur, Malaysia

## Abstract

The benzohydrazide mol­ecule in the title dihydrate, C_14_H_11_ClN_2_O_5_·2H_2_O, is non-planar, with the two aromatic rings at either side of the –C(=O)—NH—N=CH– unit forming a dihedral angle of 29.7 (2)°. The benzohydrazide mol­ecule is linked to the water mol­ecules by O—H⋯O and N—H⋯O hydrogen bonds, with other O—H⋯O hydrogen bonds leading to a layer structure.

## Related literature

For the the parent *N*′-(2-hydroxy­benzyl­idene)benzohydrazide, see: Lyubchova *et al.* (1995 ▶). For other *N*′-(2-hydr­oxy-5-nitro­benzyl­idene)benzohydrazides, see: Ali *et al.* (2005 ▶); Lyubchova *et al.* (1995 ▶); Xu & Liu (2006 ▶).
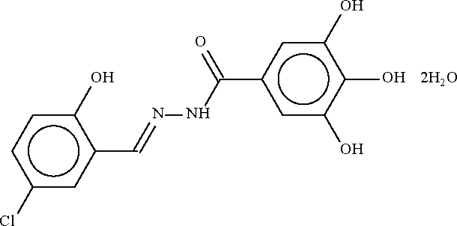


## Experimental

### 

#### Crystal data

C_14_H_11_ClN_2_O_5_·2H_2_O
*M* *_r_* = 358.73
Monoclinic, 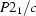

*a* = 30.5627 (12) Å
*b* = 3.7539 (2) Å
*c* = 12.8882 (5) Å
β = 90.450 (3)°
*V* = 1478.61 (11) Å^3^
*Z* = 4
Mo *K*α radiation
μ = 0.30 mm^−1^
*T* = 100 K
0.36 × 0.04 × 0.04 mm

#### Data collection

Bruker SMART APEX diffractometer
Absorption correction: multi-scan (*SADABS*; Sheldrick, 1996 ▶) *T*_min_ = 0.899, *T*_max_ = 0.988
10104 measured reflections
2623 independent reflections
1801 reflections with *I* > 2σ(*I*)
*R*_int_ = 0.097

#### Refinement

*R*[*F* ^2^ > 2σ(*F* ^2^)] = 0.071
*wR*(*F* ^2^) = 0.181
*S* = 1.07
2623 reflections
221 parameters
H-atom parameters constrained
Δρ_max_ = 0.38 e Å^−3^
Δρ_min_ = −0.49 e Å^−3^

### 

Data collection: *APEX2* (Bruker, 2008 ▶); cell refinement: *SAINT* (Bruker, 2008 ▶); data reduction: *SAINT*; program(s) used to solve structure: *SHELXS97* (Sheldrick, 2008 ▶); program(s) used to refine structure: *SHELXL97* (Sheldrick, 2008 ▶); molecular graphics: *X-SEED* (Barbour, 2001 ▶); software used to prepare material for publication: *publCIF* (Westrip, 2009 ▶).

## Supplementary Material

Crystal structure: contains datablocks global, I. DOI: 10.1107/S1600536809010812/tk2403sup1.cif

Structure factors: contains datablocks I. DOI: 10.1107/S1600536809010812/tk2403Isup2.hkl

Additional supplementary materials:  crystallographic information; 3D view; checkCIF report

## Figures and Tables

**Table 1:** Hydrogen-bond geometry (Å, °)

*D*—H⋯*A*	*D*—H	H⋯*A*	*D*⋯*A*	*D*—H⋯*A*
O1—H1⋯N1	0.84	1.89	2.631 (5)	146
O3—H3⋯O1*w*^i^	0.84	1.96	2.737 (6)	153
O4—H4⋯O1*w*^ii^	0.84	1.80	2.599 (7)	158
O5—H5⋯O2^ii^	0.84	1.93	2.765 (5)	171
O1*w*—H11⋯O3	0.83	2.28	2.969 (6)	140
O1*w*—H12⋯O4^iii^	0.84	2.07	2.900 (7)	170
O2*w*—H21⋯O1	0.84	2.15	2.946 (5)	157
O2*w*—H22⋯O2^iv^	0.84	1.97	2.808 (5)	172
N2—H2⋯O2*w*^ii^	0.88	2.03	2.882 (5)	162

Symmetry codes: (i) 
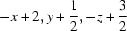
; (ii) 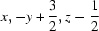; (iii) 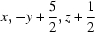; (iv) 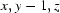.

**Fig. 1.**
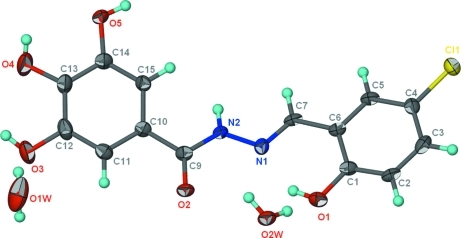
Thermal ellipsoid plot (Barbour, 2001) of C14H11ClN2O5.2H2O at the 70% probability level; hydrogen atoms are drawn as spheres of arbitrary radius.

## supplementary crystallographic information

### Experimental

5-Chloro-2-hydroxybenzaldehyde (0.31 g, 2 mmol) and 3,4,5-trihydroxybenzoylhydrazide (0.36 g, 2 mmol) were heated in ethanol (50 ml) for several hours. The solvent was removed and the product recrystallized from DMSO.

### Refinement

Carbon-bound H-atoms were placed in calculated positions [C—H 0.95 Å, *U*(H) = 1.2*U*(C)], and were included in the refinement in the riding model approximation. The amino (0.88 Å) and hydroxy H-atoms (0.84 Å) were similarly generated with U_iso_ = 1.2U_eq_(carrier atom) for N-H and U_iso_ = 1.5U_eq_(carrier atom) for O-H. The water H-atoms were placed in chemically sensible positions on the basis of possible hydrogen bonds, but were not refined; U_iso_ = 1.5U_eq_(O).

### Crystal data

**Table:**

C_14_H_11_ClN_2_O_5_·2H_2_O	*F*(000) = 744
*M**_r_* = 358.73	*D*_x_ = 1.611 Mg m^−^^3^
Monoclinic, *P*2_1_/*c*	Mo *K*α radiation, λ = 0.71073 Å
Hall symbol: -P 2ybc	Cell parameters from 1505 reflections
*a* = 30.5627 (12) Å	θ = 2.7–24.3°
*b* = 3.7539 (2) Å	µ = 0.30 mm^−^^1^
*c* = 12.8882 (5) Å	*T* = 100 K
β = 90.450 (3)°	Prism, yellow
*V* = 1478.61 (11) Å^3^	0.36 × 0.04 × 0.04 mm
*Z* = 4	

### Data collection

**Table:**

Bruker SMART APEX diffractometer	2623 independent reflections
Radiation source: fine-focus sealed tube	1801 reflections with *I* > 2σ(*I*)
graphite	*R*_int_ = 0.097
ω scans	θ_max_ = 25.0°, θ_min_ = 0.7°
Absorption correction: multi-scan (*SADABS*; Sheldrick, 1996)	*h* = −36→36
*T*_min_ = 0.899, *T*_max_ = 0.988	*k* = −4→4
10104 measured reflections	*l* = −15→14

### Refinement

**Table:**

Refinement on *F*^2^	Primary atom site location: structure-invariant direct methods
Least-squares matrix: full	Secondary atom site location: difference Fourier map
*R*[*F*^2^ > 2σ(*F*^2^)] = 0.071	Hydrogen site location: inferred from neighbouring sites
*wR*(*F*^2^) = 0.181	H-atom parameters constrained
*S* = 1.07	*w* = 1/[σ^2^(*F*_o_^2^) + (0.0537*P*)^2^ + 5.5995*P*] where *P* = (*F*_o_^2^ + 2*F*_c_^2^)/3
2623 reflections	(Δ/σ)_max_ = 0.001
221 parameters	Δρ_max_ = 0.38 e Å^−^^3^
0 restraints	Δρ_min_ = −0.49 e Å^−^^3^

### Fractional atomic coordinates and isotropic or equivalent isotropic displacement parameters (Å^2^)

**Table:**

	*x*	*y*	*z*	*U*_iso_*/*U*_eq_	
Cl1	0.53161 (4)	0.3168 (4)	0.59750 (10)	0.0244 (3)	
O1	0.67086 (11)	0.6471 (10)	0.8994 (2)	0.0243 (9)	
H1	0.6933	0.7236	0.8694	0.036*	
O2	0.79657 (11)	0.9733 (10)	0.8420 (2)	0.0232 (9)	
O3	0.94472 (12)	1.3696 (12)	0.7043 (3)	0.0349 (10)	
H3	0.9663	1.3279	0.6661	0.052*	
O4	0.94729 (12)	1.0933 (13)	0.5106 (3)	0.0395 (12)	
H4	0.9481	0.9332	0.4651	0.059*	
O5	0.87480 (11)	0.7706 (11)	0.4244 (2)	0.0245 (9)	
H5	0.8498	0.7191	0.4013	0.037*	
O1w	0.97110 (13)	0.8480 (13)	0.8663 (4)	0.0542 (14)	
H11	0.9676	0.9037	0.8040	0.081*	
H12	0.9666	1.0253	0.9042	0.081*	
O2w	0.73755 (11)	0.1847 (10)	0.9961 (2)	0.0239 (8)	
H21	0.7141	0.2633	0.9700	0.036*	
H22	0.7554	0.1438	0.9482	0.036*	
N1	0.71793 (13)	0.8677 (12)	0.7419 (3)	0.0194 (10)	
N2	0.75565 (13)	0.9864 (12)	0.6939 (3)	0.0187 (9)	
H2	0.7545	1.0529	0.6284	0.022*	
C1	0.63896 (16)	0.5781 (13)	0.8275 (4)	0.0179 (11)	
C2	0.60017 (17)	0.4266 (14)	0.8603 (4)	0.0209 (12)	
H2A	0.5965	0.3740	0.9318	0.025*	
C3	0.56699 (17)	0.3513 (14)	0.7916 (4)	0.0233 (12)	
H3A	0.5403	0.2508	0.8151	0.028*	
C4	0.57295 (16)	0.4243 (13)	0.6865 (4)	0.0189 (11)	
C5	0.61125 (16)	0.5725 (13)	0.6514 (4)	0.0193 (11)	
H5A	0.6148	0.6213	0.5797	0.023*	
C6	0.64514 (16)	0.6516 (14)	0.7219 (4)	0.0179 (11)	
C7	0.68550 (15)	0.7976 (13)	0.6815 (4)	0.0172 (11)	
H7	0.6880	0.8421	0.6092	0.021*	
C9	0.79416 (16)	1.0018 (14)	0.7457 (4)	0.0181 (11)	
C10	0.83308 (16)	1.0434 (14)	0.6800 (4)	0.0183 (11)	
C11	0.87050 (16)	1.1993 (15)	0.7207 (4)	0.0214 (12)	
H11A	0.8704	1.2939	0.7890	0.026*	
C12	0.90791 (16)	1.2173 (16)	0.6620 (4)	0.0266 (13)	
C13	0.90870 (16)	1.0734 (15)	0.5623 (4)	0.0241 (13)	
C14	0.87108 (16)	0.9157 (15)	0.5212 (4)	0.0219 (12)	
C15	0.83293 (16)	0.9046 (14)	0.5784 (4)	0.0189 (11)	
H15	0.8070	0.8046	0.5496	0.023*	

### Atomic displacement parameters (Å^2^)

**Table:**

	*U*^11^	*U*^22^	*U*^33^	*U*^12^	*U*^13^	*U*^23^
Cl1	0.0204 (7)	0.0272 (7)	0.0257 (7)	−0.0018 (6)	−0.0025 (5)	−0.0039 (6)
O1	0.026 (2)	0.031 (2)	0.0151 (17)	−0.0024 (18)	−0.0023 (15)	−0.0001 (17)
O2	0.0211 (19)	0.036 (2)	0.0122 (18)	0.0034 (17)	−0.0014 (14)	−0.0031 (16)
O3	0.022 (2)	0.049 (3)	0.034 (2)	−0.014 (2)	−0.0055 (17)	0.006 (2)
O4	0.021 (2)	0.070 (4)	0.028 (2)	−0.009 (2)	0.0042 (17)	0.011 (2)
O5	0.0199 (18)	0.042 (2)	0.0118 (17)	−0.0017 (18)	0.0023 (14)	−0.0004 (17)
O1w	0.027 (2)	0.056 (3)	0.079 (3)	0.013 (2)	0.012 (2)	0.017 (3)
O2w	0.0223 (18)	0.035 (2)	0.0139 (17)	0.0042 (17)	−0.0002 (14)	−0.0012 (17)
N1	0.015 (2)	0.024 (2)	0.019 (2)	0.0020 (18)	−0.0001 (17)	0.0006 (19)
N2	0.018 (2)	0.026 (2)	0.0121 (19)	−0.0012 (19)	0.0017 (17)	−0.0016 (19)
C1	0.023 (3)	0.016 (3)	0.014 (2)	0.002 (2)	−0.001 (2)	0.002 (2)
C2	0.027 (3)	0.022 (3)	0.013 (2)	0.002 (2)	0.005 (2)	0.000 (2)
C3	0.022 (3)	0.023 (3)	0.026 (3)	0.000 (2)	0.009 (2)	0.000 (2)
C4	0.017 (3)	0.017 (3)	0.023 (3)	0.001 (2)	0.000 (2)	−0.003 (2)
C5	0.025 (3)	0.020 (3)	0.013 (2)	0.004 (2)	0.001 (2)	0.000 (2)
C6	0.022 (3)	0.018 (3)	0.014 (2)	0.002 (2)	0.002 (2)	−0.001 (2)
C7	0.024 (3)	0.016 (3)	0.011 (2)	0.005 (2)	0.003 (2)	0.001 (2)
C9	0.021 (3)	0.018 (3)	0.015 (3)	0.001 (2)	−0.001 (2)	0.002 (2)
C10	0.019 (3)	0.023 (3)	0.013 (2)	0.003 (2)	−0.003 (2)	0.006 (2)
C11	0.022 (3)	0.024 (3)	0.018 (3)	0.001 (2)	−0.004 (2)	0.001 (2)
C12	0.019 (3)	0.036 (3)	0.025 (3)	−0.007 (2)	−0.006 (2)	0.013 (3)
C13	0.017 (3)	0.035 (3)	0.020 (3)	−0.001 (2)	0.000 (2)	0.009 (2)
C14	0.024 (3)	0.029 (3)	0.013 (2)	0.000 (2)	−0.002 (2)	0.007 (2)
C15	0.018 (3)	0.022 (3)	0.017 (2)	−0.001 (2)	−0.002 (2)	0.002 (2)

### Geometric parameters (Å, °)

**Table:**

Cl1—C4	1.747 (5)	C1—C6	1.403 (6)
O1—C1	1.365 (6)	C2—C3	1.371 (7)
O1—H1	0.8400	C2—H2A	0.9500
O2—C9	1.247 (6)	C3—C4	1.395 (7)
O3—C12	1.371 (6)	C3—H3A	0.9500
O3—H3	0.8400	C4—C5	1.376 (7)
O4—C13	1.361 (6)	C5—C6	1.403 (7)
O4—H4	0.8400	C5—H5A	0.9500
O5—C14	1.366 (6)	C6—C7	1.450 (7)
O5—H5	0.8400	C7—H7	0.9500
O1w—H11	0.8347	C9—C10	1.474 (7)
O1w—H12	0.8378	C10—C11	1.384 (7)
O2w—H21	0.8421	C10—C15	1.408 (7)
O2w—H22	0.8400	C11—C12	1.377 (7)
N1—C7	1.283 (6)	C11—H11A	0.9500
N1—N2	1.386 (6)	C12—C13	1.395 (8)
N2—C9	1.350 (6)	C13—C14	1.394 (7)
N2—H2	0.8800	C14—C15	1.385 (7)
C1—C2	1.384 (7)	C15—H15	0.9500
			
C1—O1—H1	109.5	C1—C6—C7	122.9 (4)
C12—O3—H3	109.5	C5—C6—C7	118.3 (4)
C13—O4—H4	109.5	N1—C7—C6	121.0 (4)
C14—O5—H5	109.5	N1—C7—H7	119.5
H11—O1w—H12	110.0	C6—C7—H7	119.5
H21—O2w—H22	109.0	O2—C9—N2	122.2 (4)
C7—N1—N2	115.9 (4)	O2—C9—C10	122.6 (4)
C9—N2—N1	121.2 (4)	N2—C9—C10	115.1 (4)
C9—N2—H2	119.4	C11—C10—C15	120.3 (5)
N1—N2—H2	119.4	C11—C10—C9	119.7 (4)
O1—C1—C2	118.6 (4)	C15—C10—C9	119.9 (4)
O1—C1—C6	121.4 (4)	C12—C11—C10	120.0 (5)
C2—C1—C6	120.0 (4)	C12—C11—H11A	120.0
C3—C2—C1	121.2 (5)	C10—C11—H11A	120.0
C3—C2—H2A	119.4	O3—C12—C11	119.0 (5)
C1—C2—H2A	119.4	O3—C12—C13	120.5 (5)
C2—C3—C4	119.0 (5)	C11—C12—C13	120.5 (5)
C2—C3—H3A	120.5	O4—C13—C14	123.5 (5)
C4—C3—H3A	120.5	O4—C13—C12	116.8 (5)
C5—C4—C3	121.1 (5)	C14—C13—C12	119.7 (5)
C5—C4—Cl1	119.4 (4)	O5—C14—C15	123.4 (4)
C3—C4—Cl1	119.5 (4)	O5—C14—C13	116.2 (4)
C4—C5—C6	119.9 (4)	C15—C14—C13	120.3 (5)
C4—C5—H5A	120.0	C14—C15—C10	119.2 (4)
C6—C5—H5A	120.0	C14—C15—H15	120.4
C1—C6—C5	118.8 (5)	C10—C15—H15	120.4
			
C7—N1—N2—C9	−166.9 (5)	N2—C9—C10—C11	154.5 (5)
O1—C1—C2—C3	−180.0 (5)	O2—C9—C10—C15	148.7 (5)
C6—C1—C2—C3	−1.1 (8)	N2—C9—C10—C15	−29.5 (7)
C1—C2—C3—C4	1.0 (8)	C15—C10—C11—C12	−0.4 (8)
C2—C3—C4—C5	−0.6 (8)	C9—C10—C11—C12	175.6 (5)
C2—C3—C4—Cl1	178.0 (4)	C10—C11—C12—O3	−179.3 (5)
C3—C4—C5—C6	0.2 (8)	C10—C11—C12—C13	−1.2 (8)
Cl1—C4—C5—C6	−178.4 (4)	O3—C12—C13—O4	−0.1 (8)
O1—C1—C6—C5	179.6 (5)	C11—C12—C13—O4	−178.2 (5)
C2—C1—C6—C5	0.7 (7)	O3—C12—C13—C14	179.2 (5)
O1—C1—C6—C7	1.2 (8)	C11—C12—C13—C14	1.1 (8)
C2—C1—C6—C7	−177.7 (5)	O4—C13—C14—O5	1.3 (8)
C4—C5—C6—C1	−0.3 (7)	C12—C13—C14—O5	−178.0 (5)
C4—C5—C6—C7	178.2 (5)	O4—C13—C14—C15	179.9 (5)
N2—N1—C7—C6	176.3 (4)	C12—C13—C14—C15	0.6 (8)
C1—C6—C7—N1	−0.1 (8)	O5—C14—C15—C10	176.3 (5)
C5—C6—C7—N1	−178.5 (5)	C13—C14—C15—C10	−2.2 (8)
N1—N2—C9—O2	−13.1 (8)	C11—C10—C15—C14	2.1 (7)
N1—N2—C9—C10	165.1 (4)	C9—C10—C15—C14	−173.9 (5)
O2—C9—C10—C11	−27.4 (8)		

### Hydrogen-bond geometry (Å, °)

**Table:**

*D*—H···*A*	*D*—H	H···*A*	*D*···*A*	*D*—H···*A*
O1—H1···N1	0.84	1.89	2.631 (5)	146
O3—H3···O1w^i^	0.84	1.96	2.737 (6)	153
O4—H4···O1w^ii^	0.84	1.80	2.599 (7)	158
O5—H5···O2^ii^	0.84	1.93	2.765 (5)	171
O1w—H11···O3	0.83	2.28	2.969 (6)	140
O1w—H12···O4^iii^	0.84	2.07	2.900 (7)	170
O2w—H21···O1	0.84	2.15	2.946 (5)	157
O2w—H22···O2^iv^	0.84	1.97	2.808 (5)	172
N2—H2···O2w^ii^	0.88	2.03	2.882 (5)	162

Symmetry codes: (i) −*x*+2, *y*+1/2, −*z*+3/2; (ii) *x*, −*y*+3/2, *z*−1/2; (iii) *x*, −*y*+5/2, *z*+1/2; (iv) *x*, *y*−1, *z*.
